# High genetic diversity and stable Pleistocene distributional ranges in the widespread Mexican red oak *Quercus castanea* Née (1801) (Fagaceae)

**DOI:** 10.1002/ece3.6189

**Published:** 2020-03-24

**Authors:** Juan Manuel Peñaloza‐Ramírez, Hernando Rodríguez‐Correa, Antonio González‐Rodríguez, Víctor Rocha‐Ramírez, Ken Oyama

**Affiliations:** ^1^ Escuela Nacional de Estudios Superiores (ENES) Unidad Morelia Universidad Nacional Autónoma de México (UNAM) Morelia México; ^2^ Instituto de Investigaciones en Ecosistemas y Sustentabilidad UNAM Morelia México

**Keywords:** genetic variation, historical demography, Mexican Highlands, neotropical trees, oaks, phylogeography

## Abstract

The Mexican highlands are areas of high biological complexity where taxa of Nearctic and Neotropical origin and different population histories are found. To gain a more detailed view of the evolution of the biota in these regions, it is necessary to evaluate the effects of historical tectonic and climate events on species. Here, we analyzed the phylogeographic structure, historical demographic processes, and the contemporary period, Last Glacial Maximum (LGM) and Last Interglacial (LIG) ecological niche models of *Quercus castanea*, to infer the historical population dynamics of this oak distributed in the Mexican highlands. A total of 36 populations of *Q. castanea* were genotyped with seven chloroplast microsatellite loci in four recognized biogeographic provinces of Mexico: the Sierra Madre Occidental (western mountain range), the Central Plateau, the Trans‐Mexican Volcanic Belt (TMVB, mountain range crossing central Mexico from west to east) and the Sierra Madre del Sur (SMS, southern mountain range). We obtained standard statistics of genetic diversity and structure and tested for signals of historical demographic expansions. A total of 90 haplotypes were identified, and 29 of these haplotypes were restricted to single populations. The within‐population genetic diversity was high (mean *h*_S_ = 0.72), and among‐population genetic differentiation showed a strong phylogeographic structure (*N*_ST_ = 0.630 > *G*_ST_ = 0.266; *p* < .001). Signals of demographic expansion were identified in the TMVB and the SMS. The ecological niche models suggested a considerable percentage of stable distribution area for the species during the LGM and connectivity between the TMVB and the SMS. High genetic diversity, strong phylogeographic structure, and ecological niche models suggest in situ permanence of *Q. castanea* populations with large effective population sizes. The complex geological and climatic histories of the TMVB help to explain the origin and maintenance of a large proportion of the genetic diversity in this oak species.

## Graphical abstract

High genetic diversity in Mexican oaks. High phylogeographic structure in a widespread oak species.

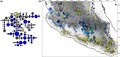

## 1 INTRODUCTION

The Mexican Highlands encompass the main mountain systems of Mexico (i.e., Sierra Madre Occidental, Sierra Madre Oriental, Trans‐Mexican Volcanic Belt, Sierra Madre del Sur and Altos de Chiapas) and are located between the Nearctic and Neotropical regions. Biogeographically, the Mexican Highlands comprise most of the Mexican Transition Zone which also includes the Central American mountains forming the Chiapas Highlands province (Morrone, 2014). In the Mexican Transition zone, key evolutionary processes have been studied such as species diversification and historical demography including the role of paleoclimatic and geological events in the evolutionary history of the species (Mastretta‐Yanes, Moreno‐Letelier, Piñero, Jorgensen, & Emerson, 2015).

The Mexican Transition Zone is characterized by an impressive biological and physical complexity in terms of biogeographical and macroecological patterns (Morrone, 2010; Ríos‐Muñoz & Navarro‐Sigüenza, 2012; Sosa, Ornelas, Ramírez‐Barahona, & Gándara, 2016), geological evolution (De Cserna, 1989; Ferrari, Orozco‐Esquivel, Manea, & Manea, 2012), and paleoclimatic dynamics (Caballero, Lozano‐García, Ortega‐Guerrero, & Correa‐Metrio, 2019; Caballero‐Rodríguez et al., 2018). Therefore, the study of the evolution of the biota in the Mexican Transition Zone requires the consideration of both biotic and abiotic factors, as the phylogeographic approaches do. Even though initial syntheses concerning multitaxa phylogeographic patterns in the Mexican Transition Zone suggest some common responses of species to geographic barriers and historical climate changes (Ramírez‐Barahona, & Eguiarte, 2013; Ornelas et al., 2013), we are still far from attaining a detailed view of the processes that have shaped the diversity and evolution of the species in the region.

The most important facts that phylogeographic studies have shown for species in the Mexican Transition Zone include pre‐Quaternary divergence times, east–west phylogeographical breaks in the Trans‐Mexican Volcanic Belt that are incongruent with geographic distance or topography, and north–south phylogeographical breaks within the Sierra Madre Occidental (Gutiérrez‐García & Vázquez‐Domínguez, 2013; Mastretta‐Yanes et al., 2015; Ornelas et al., 2013 and references therein). However, shortcomings such as differences in sampling schemes among studies, circumstantial choice of the studied taxa and the prevalence of both allopatric and parapatric genetic divergence (Mastretta‐Yanes et al., 2015) have made the identification of general phylogeographic patterns a difficult task.

Oaks (*Quercus*: Fagaceae) have been frequently used to study the geographic distribution of genetic diversity from a historical point of view in the Nearctic and Neotropical regions (Ashley, Backs, Kindsvater, & Abraham, 2018; Cavender‐Bares et al., 2015; Gugger & Cavender‐Bares, 2013; Magni, Ducousso, Caron, Petit, & Kremer, 2005; Marsico, Hellmann, & Romero‐Severson, 2009; Rodríguez‐Correa, Oyama, Quesada, Fuchs, & González‐Rodríguez, 2018; Rodríguez‐Gómez, Oyama, & Ochoa‐Orozco, 2018). Recently, several authors described a well‐founded diversification scenario for American oaks (Hipp et al., 2014, 2018, 2019). These studies have shown that the species diversity of *Quercus* in the Americas is the product of a complex sympatric parallel diversification of the two main sections within the genus, which increased as the species reached the Mexican highlands where new ecological niches were rapidly colonized (Hipp et al., 2018). These new results have helped to establish the genus *Quercus* as a model clade to integrate ecological and evolutionary processes (Cavender‐Bares, 2019).

On this basis, for this study we chose *Quercus castanea* Neé (1801) as a focal species. This species has high ecological importance and has been intensively studied for gene flow estimation in fragmented landscapes (Herrera‐Arroyo et al., 2013; Oyama et al., 2017) and community genetics (Tovar‐Sánchez et al., 2013). Additionally, *Q. castanea* has one of the broadest geographical and altitudinal distributions among Mexican oaks. Therefore, focusing on this species may enable us to study historical processes through a large area that experienced a wide range of tectonic and paleoclimate events and shed light into the complex evolutionary processes of Nearctic and Neotropical biotas.

Specifically, the objectives of the present study were to (a) determine the patterns of genetic diversity and differentiation of populations of *Q. castanea* across its entire geographical range using simple sequence repeats of the chloroplast DNA (cpSSR), (b) test for signals of historical demographic expansions and to determine the timing of the population expansion, (c) contrast the conclusions derived from genetic data with models of the contemporary period, LGM and LIG ecological niche of *Q. castanea*, and (d) contrast the phylogeographic patterns revealed in this study with the regionalization of the biogeographic provinces based on the physiographic features.

## 2 MATERIALS AND METHODS

### 2.1 Study species and sampling procedure

**Figure 1:**
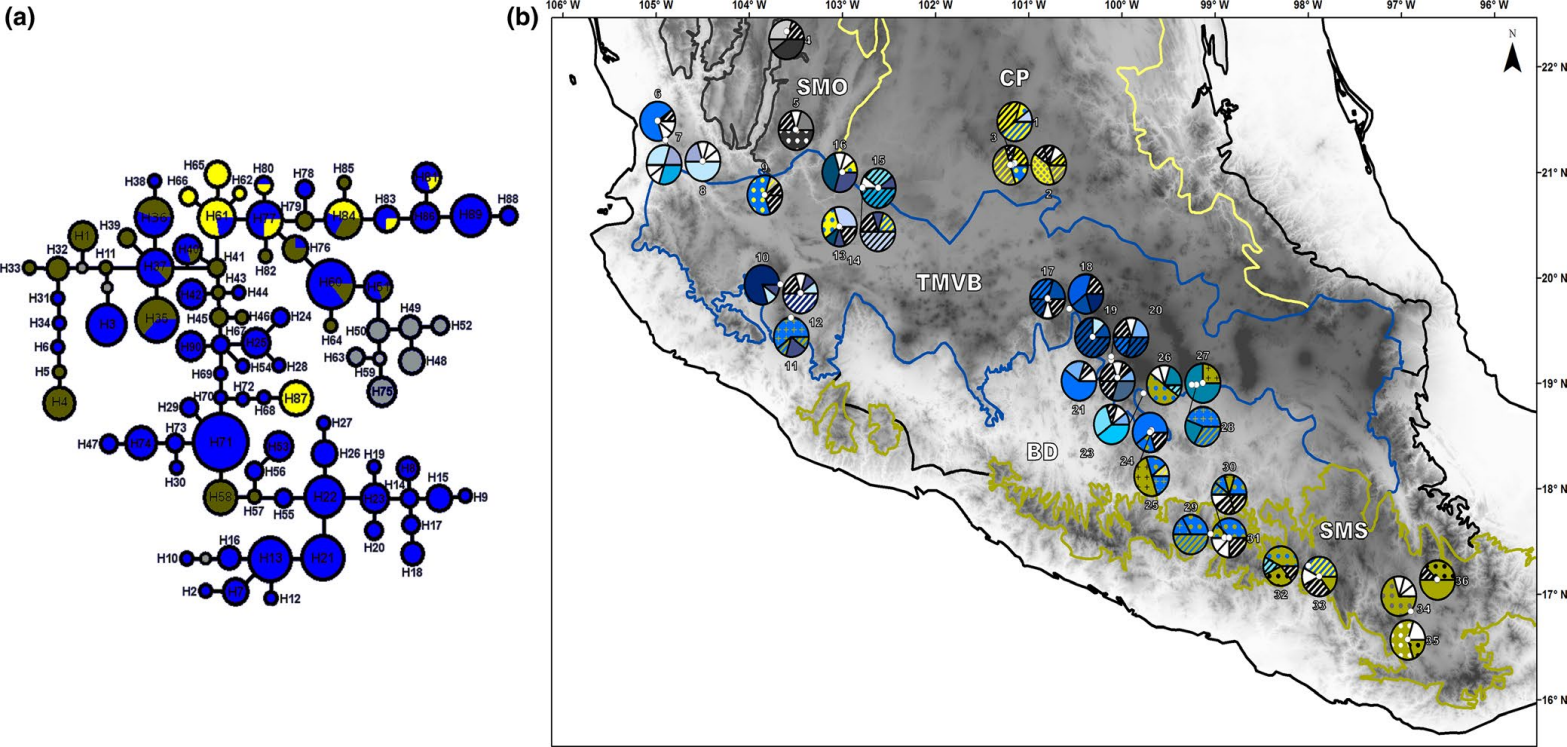
(a) Median‐joining haplotype network for the 90 haplotypes identified in 36 populations of *Q. castanea* in Mexico. Each circle represents an individual haplotype, and circle size is proportional to the frequency of the haplotype. Different colors indicate the presence of the haplotypes in the four biogeographic regions: in gray haplotypes present in the SMO, in blue haplotypes present in the TMVB, in yellow haplotypes present in the CP, and in green haplotypes present in the SMS. (b) Haplotype frequencies in 36 sampled populations of *Quercus castanea* in four morphotectonic and biogeographic provinces of Mexico: Sierra Madre Occidental (SMO gray lines), Central Plateau (CP yellow lines), Trans‐Mexican Volcanic Belt (TMVB blue lines), and Sierra Madre del Sur (SMS green lines). Unique and restricted haplotypes are presented in white and black‐white striped colors, respectively. Elevation is represented in a gray scale from lowlands (light gray) to highlands (dark gray)

*Quercus castanea* belongs to the section *Lobatae* (Valencia‐Á., 2004). This species is a moderately large forest tree reaching 18 m in height. Populations are found between 1,100 and 2,600 m of elevation, mostly in oak forests and pine–oak forests but occasionally in association with tropical dry forest elements at lower altitudes. This tree grows in mountainous areas with warm to temperate humid climates, with average annual temperatures ranging from 10 to 26°C (Kappelle, 2006) and where the rainy season occurs in the warm season of the year (Rzedowski, 1978). The species has a flowering period from April to May and is wind‐pollinated. Acorns mature from July to November, and dispersal occurs by gravity, birds, and squirrels (Valencia‐A., 1995). We sampled 36 populations with approximately 10 individuals per population across the *Q. castanea* geographical distribution in Mexico (Table 1, Figure 1). Within populations, individuals were randomly selected with at least 10 m of separation between consecutive samples. From each tree, five young intact leaves were collected and stored at −80°C for molecular analysis, and a branch was pressed for further confirmation of species identity.

**Table 1:** Geographical location and estimates of genetic diversity for 36 populations of *Quercus castanea* in four biogeographic provinces of Mexico

Population/Region ID	*n*	Latitude	Longitude	Elevation (m)	Allelic richness	Allelic richness rarefacted	*h_s_*	*D* ^2^_SH_
1	10	21.08	−101.15	2,452	4	2.95	0.73	0.23
2	10	21.07	−101.18	2,150	6	3.75	0.84	0.09
3	10	21.07	−101.20	2,558	4	3.05	0.73	0.33
**R1**	**CP**				10	8.44	0.87	0.11
4	10	22.33	−103.60	1,732	4	3.17	0.78	0.05
5	10	21.40	−103.50	1,916	4	3.05	0.73	0.07
**R2**	**SMO**				7	7.00	0.87	0.06
6	10	21.48	−104.98	1,279	4	2.50	0.53	0.05
7	10	21.30	−104.90	1,192	5	3.61	0.84	0.04
8	10	21.10	−104.50	1,065	5	3.27	0.76	0.05
9	10	20.78	−103.83	1,912	4	2.78	0.64	0.13
10	10	19.93	−103.67	2,200	3	2.00	0.38	0.38
11	10	19.62	−103.55	2,487	4	2.78	0.64	0.22
12	10	19.85	−103.45	1,910	5	3.27	0.76	0.20
13	10	20.48	−103.03	2,273	5	3.61	0.84	0.04
14	10	20.85	−102.78	2,115	4	3.05	0.73	0.12
15	10	20.85	−102.62	1,889	4	2.91	0.71	0.15
16	10	21.00	−103.00	1,907	5	3.39	0.80	0.10
17	10	19.80	−100.80	1,970	5	3.75	0.87	0.06
18	10	19.70	−100.57	2,080	4	3.31	0.80	0.10
19	8	19.43	−100.32	2,530	3	2.25	0.46	0.10
20	10	19.25	−100.12	1,908	5	3.75	0.87	0.44
21	10	19.22	−100.12	1,901	4	2.78	0.64	0.12
22	10	19.05	−100.05	1,907	6	3.97	0.89	0.25
23	10	18.90	−99.77	2,150	5	3.39	0.80	0.19
24	10	18.53	−99.70	2,190	3	2.56	0.62	0.68
25	10	18.55	−99.68	2,001	4	3.05	0.73	0.13
26	10	18.98	−99.25	2,189	5	3.27	0.76	0.13
27	8	19.00	−99.13	2,010	2	1.89	0.43	0.28
28	9	18.98	−99.20	1,980	3	2.78	0.72	0.45
**R3**	**TMVB**				62	15.96	0.97	0.01
29	6	17.57	−99.05	1,871	3	2.83	0.73	0.64
30	10	17.53	−98.90	2,028	7	4.33	0.93	0.08
31	8	17.53	−98.85	1,890	5	3.75	0.86	0.20
32	7	17.27	−98.00	1,960	4	3.38	0.81	0.12
33	7	17.17	−97.88	2,485	4	3.38	0.81	0.35
34	10	16.83	−96.90	1,748	4	2.50	0.53	0.38
35	5	16.57	−96.93	1,845	3	3.00	0.70	0.23
36	8	17.13	−96.62	2,430	3	2.61	0.68	0.19
**R4**	**SMS**				25	13.53	0.95	0.02

Note

*n*, sample size; *h*_S_, within‐population genetic diversity; *D* ^2^sh, mean pairwise genetic distance among individuals within a population under a stepwise mutation model. Allelic richness is reported after a rarefaction analysis to standardize for unequal sample sizes.

Abbreviations: CP, Central Plateu; SMO, Sierra Madre Oriental; SMS, Sierra Madre del Sur; TMVB, Trans‐Mexican Volcanic Belt.

### 2.2 Molecular methods

Genomic DNA was extracted from 100 mg of leaf material using the modified CTAB protocol of Lefort and Douglas (1999). Seven cpDNA microsatellite loci were selected and amplified in multiplex polymerase chain reactions (PCR). Two groups of primers were arranged according to allele size and fluorescent labels. The first group was formed by the primer pairs UDT1, UDT3, UCD5, UKK3, and UKK4 previously designed by Deguilloux, Dumolin‐Lapègue, and Gielly (2003). The second group of primers included CMCS6 and CMCS10 previously designed by Sebastiani, Carnevale, and Vendramin (2004). PCRs were performed using the QIAGEN Multiplex PCR kit (QIAGEN) in a volume of 5 µl containing 1X Multiplex PCR Master Mix, 2 µM each primer, dH_2_0, and 20 ng template DNA. The thermal cycling conditions consisted of an initial denaturation step for 15 min at 95°C, followed by 40 cycles, each at 95°C for 1 min, annealing at 50°C for 1 min, and extension at 72°C for 2 min. A final extension at 72°C for 10 min was included. Multiplex PCR products were combined with a GeneScan‐500 LIZ size standard and then run in an ABI‐PRISM 3100‐Avant sequencer (Applied Biosystems). Fragments were analyzed and sized with the Peak Scanner program 1.0 (Applied Biosystems).

### 2.3 Genetic diversity

Genetic diversity parameters were estimated for each population and for four regions commonly recognized as morphotectonic and biogeographic units for Mexico (the Sierra Madre Occidental, the Trans‐Mexican Volcanic Belt, the Central Plateau and the Sierra Madre del Sur) (Ferrusquía‐Villafranca, 1993; Morrone, 2005, 2014). Population‐level and regional haplotype richness (corrected for unequal sample sizes with rarefaction), number of exclusive haplotypes and gene diversity (*h_S_*) were calculated using SPAGeDi 1.1 (Hardy & Vekemans, 2002). *D* ^2^_SH_, which is the mean pairwise genetic distance among individuals within a population under a stepwise mutation model (Goldstein, Ruiz Bares, Cavalli‐Sforzaf, & Feldman, 1995), was calculated using the LMSE program (Navascués, Hardy, & Burgarella, 2009). To assess the patterns of the geographical distribution of genetic variation in *Q. castanea*, genetic diversity indexes were correlated with elevation, latitude, and longitude using a correlation test implemented in R 3.2.2 (R Core Team, 2017). To depict genealogical relationships among haplotypes, a median‐joining network was constructed using the software NETWORK 4.5 (Bandelt, Forster, Sykes, & Richards, 1995). This method combines the topology of a minimum spanning tree with a maximum parsimony search (Polzin & Daneshmand, 2003) to identify and remove unnecessary links between haplotypes.

### 2.4 Genetic and phylogeographic structure

Tests of population genetic structure were performed using analyses of molecular variance (AMOVA) with ARLEQUIN 3.5 (Excoffier, Laval, & Schneider, 2007) to obtain estimates of both *F*_ST_ (based on the infinite alleles mutation model, IAM) and *R*_ST_ (based on the stepwise mutation model, SMM). We also performed a hierarchical grouping to obtain the variance components of genetic variation among groups, among populations within groups, and within populations. The categories were defined by the four above‐mentioned biogeographic provinces. The significance of partitions was tested using 10^4^ permutations.

Phylogeographic structure was determined by comparing the differentiation coefficients for unordered and ordered alleles (*N*_ST_ and *G*_ST_, respectively) in SPAGeDi 1.1 (Hardy & Vekemans, 2002). If *N*_ST_, which considers the genetic differences among the haplotypes, is significantly higher than *G*_ST_, then there is phylogeographic structure in the populations (Pons & Petit, 1996). To determine whether phylogeographic structure was determined by regional diversity, differences between mean *N*_ST_ and mean *G*_ST_ were calculated through regular distance intervals. This analysis was implemented in SPAGeDi 1.1 (Hardy & Vekemans, 2002) using four distance intervals. Geographic intervals were defined by calculating a distance that homogenizes the number of populations represented in each interval.

We analyzed the spatial distribution of genetic variation and identified genetic discontinuities between *Q. castanea* populations using GENELAND 4.0.7 (Guillot, Renaud, Ledevin, Michaux, & Claude, 2012), which considers genetic structure together with the geographic location of populations using a model of spatial Bayesian clustering under a MCMC method, to assign individuals into genetic clusters (Guillot et al., 2012; Safner, Miller, McRae, Fortin, & Manel, 2011). GENELAND was implemented using a number of potential spatial clusters (*K*) ranging from two to 35. The analyses were set up considering ten independent runs, correlated allele frequencies, one million generations with a thinning value of 100 and a burn‐in value of 1,000. Finally, the distribution of GENELAND spatial clusters was mapped using ArcMap 10.3 (ESRI Inc.).

### 2.5 Historical population demography

Tests for population expansions were performed at the population and regional levels using *F*_S_ (Fu, 1997) and mismatch distributions (Rogers & Harpending, 1992). The *F*_S_ statistic evaluates the probability of observing a random neutral sample with a number of alleles similar to or smaller than the observed value, given the observed number of pairwise differences and taken as an estimator of Θ (Fu, 1997). Statistically significant negative *F*_S_ values result from an excess of recent mutations (i.e., rare haplotypes) and suggest population expansion or purifying selection. In turn, positive *F_S_* values indicate a recent population bottleneck or the action of balancing selection. Simulations have shown that *F_S_* is a more precise indicator of population historical demography than Tajima's *D* (Ramos‐Onsins & Rozas, 2002).

Expanding populations are also expected to have unimodal distributions for differences in repeat number in pairwise comparisons among individuals in a sample (i.e., the mismatch distribution), while in stationary populations, the distribution is predicted to be ragged and multimodal (Rogers & Harpending, 1992). To use ARLEQUIN 3.5 for these analyses, the cpSSR data were binary coded following Navascués and Emerson (2005) and Navascués et al. (2009). The number of repeats was coded as “1,” and shorter alleles were coded filling the differences in repeats with “0.” The significance of *F*_S_ values was evaluated with 10^4^ data bootstraps (Excoffier et al., 2007). For the mismatch distributions, the raggedness index of Harpending (1994) was used to evaluate the goodness of fit of the observed distributions to those distributions expected under the model of population expansion.

For populations and biogeographical regions with significant *F_S_* negative values and nonsignificant raggedness index, we estimated the time to the population expansion (*τ* = 2*lµt*) and the initial and present effective population sizes scaled by mutation rate (Θ_0_ = 2*µN*_0_ and Θ_1_ = 2*µN*_1_), where *µ* represents the mutation rate, *t* is the number of generations since the population expansion occurred, *l* is the number of loci evaluated, and *N_0_* and *N_1_* are the previous effective population sizes and the sizes after the expansion, respectively (Rogers & Harpending, 1992). The pseudolikelihood method (Navascués et al., 2009) implemented in the LMSE program was used to estimate these parameters. This method takes homoplasy, which is common for cpSSRs, into account in the calculations. Since no reliable estimates of the mutation rate for cpSSRs have been reported, the translation of these values into years is subjected to considerable uncertainty. However, mutation rates in the range of 10^–5^ to 10^–4^ mutations per generation per locus have been usually assumed in the literature, as well as generation times for trees between 25 and 100 years (Heuertz, Teufel, & González‐Martínez, 2010; Navascués et al., 2009). Hence, we used those values of mutation and generation times to calculate *t.*

### 2.6 Ecological niche modeling

A total of 520 *Q. castanea* occurrence records were compiled from herbarium specimens deposited at the Herbario Nacional de México (MEXU), the TROPICOS database (Missouri Botanical Garden), and the Global Biodiversity Information Facility database (GBIF; http://www.data.gbif.org/). Records that did not match the natural distribution of the species reported by Valencia‐Á (2004) were eliminated from the dataset. To reduce spatial autocorrelation in climatic data, a 0.1° filter was applied to the initial set of occurrences, resulting in a total of 298 *Q. castanea* occurrence records to run the model. The maximum entropy algorithm implemented in MAXENT 3.3.3k (Phillips, Anderson, & Schapire, 2006) was used to generate a contemporary period niche model and to project it into two Last Glacial Maximum (LGM; ~21 ka) and Last Interglacial (LIG; ~120 ka) climatic scenarios using data from environmental variables (Hijmans, Cameron, Parra, Jones, & Jarvis, 2005).

Contemporary climate layers (average of the 1960–1990 period) were downloaded from the WorldClim 1.4 database (Hijmans et al., 2005; http://www.worldclim.org) with a 30 arc seconds resolution. The LGM climate layers were obtained from two past climate scenarios developed by the Coupled Model Intercomparison Project Phase 5 (Meehl & Bony, 2011): the Community Climate System Model (CCSM) (Collins et al., 2006) version 4.0 and the Model for Interdisciplinary Research on Climate (Hasumi & Emori, 2004) MIROC‐ESM (Watanabe et al., 2011), both with a 2.5 arc minutes resolution which are also available on the WorldClim webpage. Climate layers for the LIG developed by Otto‐Bliesner et al. (2008) with a 30 arc seconds resolution were downloaded from the WorldClim webpage. Finally, elevation was considered for the different time periods using the ETOPO1 Global Relief Model developed by Amante and Eakins (2009) with a 2.5 arc minutes resolution. In order to include elevation for the MAXENT modeling two layers were used, the first one with a 2.5 arc minutes for the LGM projections, and a second one rescaled to a 30 arc seconds resolution for the contemporary and LIG periods, generated by interpolating the original layer using the *spline* function in ARCMAP 10.3 (Esri Inc.).

To avoid redundancy among variables, the initial set of 20 variables was reduced to a group of seven variables using a correlation matrix implemented with the *corrplot* (Wei & Simko, 2017) function in R 3.2.2 (R Core Team, 2017). When combinations showed *r* values over .7, the global variables were retained over the particular ones (e.g., annual mean temperature was chosen over mean temperature of the wettest month). In addition to elevation, the bioclimatic variables retained represent annual trends (mean annual temperature, annual precipitation), seasonality (temperature seasonality, precipitation seasonality, annual range in temperature), and extreme or limiting environmental factors (precipitation of the driest month).

As conventionally used, a niche model was produced under contemporary climatic conditions and then projected into the past climatic scenarios (Nogués‐Bravo, 2009). To run the model, a subset of 70% of *Q. castanea* records were used as training data, and the remaining 30% occurrences were used to validate it. As a threshold‐independent method for model validation, we used the area under the receiver operating characteristic curve (AUC) (Fielding & Bell, 1997). Fifty independent runs using bootstrap replication were performed for each model to assure convergence on similar solutions. A jackknife test for the model in the contemporary period was used to measure the relative importance of each climatic variable on the occurrence prediction.

To evaluate whether the models suggest changes in the distribution of *Q. castanea* between the LIG, LGM, and the contemporary periods, we defined the presence of the species using the 10 percentile training presence threshold rule (Phillips et al., 2006). In order to identify the areas within the distribution of *Q. castanea* that have remained stable through the different time periods (LIG, LGM and contemporary), binomial outputs calculated using the above‐mentioned threshold rule were summed using the *raster calculator* tool in ARCMAP 10.4 (ESRI Inc.) and the extent of the stable areas was compared to the entire area of the contemporary distribution model.

## 3 RESULTS

### 3.1 Genetic diversity and structure

A total of 90 haplotypes were found in the 36 sampled populations of *Q. castanea* (Table 1; Figure 1) with 29 singletons. The number of haplotypes per population ranged from two to seven. At the level of biogeographic provinces, the Central Plateau (Table 1; R1, populations 1–3) had a total of 10 haplotypes, the Sierra Madre Occidental (Table 1; R2, populations 4–5) had seven haplotypes, the Trans‐Mexican Volcanic Belt (Table 1; R3, populations 6–28) had 62 haplotypes, and the Sierra Madre del Sur (Table 1; R4, populations 29–36) had 25 haplotypes. Haplotype richness and genetic diversity (*h*_S_) were higher in the Trans‐Mexican Volcanic Belt (15.96 and 0.97, respectively) and the Sierra Madre del Sur (13.53, 0.95) than in the Central Plateau (8.44, 0.87) and the Sierra Madre Occidental (7.0, 0.87) (Table 1). Genetic diversity parameters were not correlated with latitude, longitude or altitude of the populations, except for *D* ^2^_SH_, which increased with altitude (*r* = .43; *p* = .008) and longitude (*r* = .41; *p* = .012) and decreased with latitude (*r* = −.44; *p* = .006).

The mean within‐population genetic diversity (*h*_S_) was 0.725 (0.022), and the total diversity (*h*_T_) was 0.989 (0.003). The analysis of molecular variance (AMOVA) showed that most of the variation occurred among populations under the IAM and SMM (59.60% and 64.81%, respectively). The hierarchical AMOVA showed that when distances among haplotypes are not taken into account (IAM), most of the differentiation resides among populations within regions (35.39%), followed by among the four regions (35.13%), and within populations (29.28%; Table 2). In contrast, if distances among haplotypes are considered (SMM), the genetic differentiation among populations within regions is 46.33%, while variation residing within populations is 37.22% and variation among the four regions is 16.45% (Table 2).

**Table 2:** Analysis of molecular variance (AMOVA) using *F*_ST_ and *R*_ST_ for 36 populations of *Quercus castanea*

Source of variation	*df*	ss	Variance components	Percentage of variation	Fixation index
*F*_ST_					
Among populations	35	504.45	1.44	59.60	
Within populations	300	292.86	0.97	40.40	
Total	335	797.31	2.41		
Among groups	3	110.84	0.43	16.45	Φ_CT_ = 0.16*
Among populations within groups	32	393.61	1.21	46.33	Φ_SC_ = 0.55*
Within populations	300	292.86	0.97	37.22	Φ_ST_ = 0.16*
Total	335	797.31	2.62		
*R*_ST_					
Among populations	35	6,538.02	18.92	64.81	
Within populations	300	3,082.04	10.27	35.19	
Total	335	9,620.06	29.19		
Among groups	3	2,482.88	12.32	35.13	Φ_CT_ = 0.35*
Among populations within groups	32	4,053.14	12.48	35.59	Φ_SC_ = 0.54*
Within populations	300	3,082.04	10.27	29.28	Φ_ST_ = 0.70*
Total	335	9,620.06	35.09		

Note

Hierarchical AMOVA groups defined according to biogeographic provinces. Asterisks indicate statistically significant values (*p* < .001). Tests were based on 10 000 random permutations.

The network of the 90 haplotypes identified in *Q. castanea* is shown in Figure 1a. In most cases, haplotypes were separated by one mutational step. Some groups of closely related haplotypes were restricted to specific geographical regions. This restriction was particularly clear for the gray group haplotypes (Figure 1a), which were the only haplotypes present in the Sierra Madre Occidental and were exclusive to this region. In the case of the Central Plateau, four of the haplotypes were geographically restricted to the region (solid yellow haplotypes in Figure 1a), six haplotypes were shared with the Trans‐Mexican Volcanic Belt (H61, H77, H80, H81, H83), and one haplotype was shared by both the Trans‐Mexican Volcanic Belt and the Sierra Madre del Sur (H84). Within the Sierra Madre del Sur, 17 haplotypes were geographically restricted (solid green haplotypes in Figure 1a), seven haplotypes (H35, H36, H37, H40, H51, H60, and H76) were shared by the Sierra Madre del Sur and the Trans‐Mexican Volcanic Belt. Most of the haplotypes were present in the Trans‐Mexican Volcanic Belt (solid blue haplotypes in Figure 1a).

### 3.2 Phylogeographic structure

**Figure 2:**
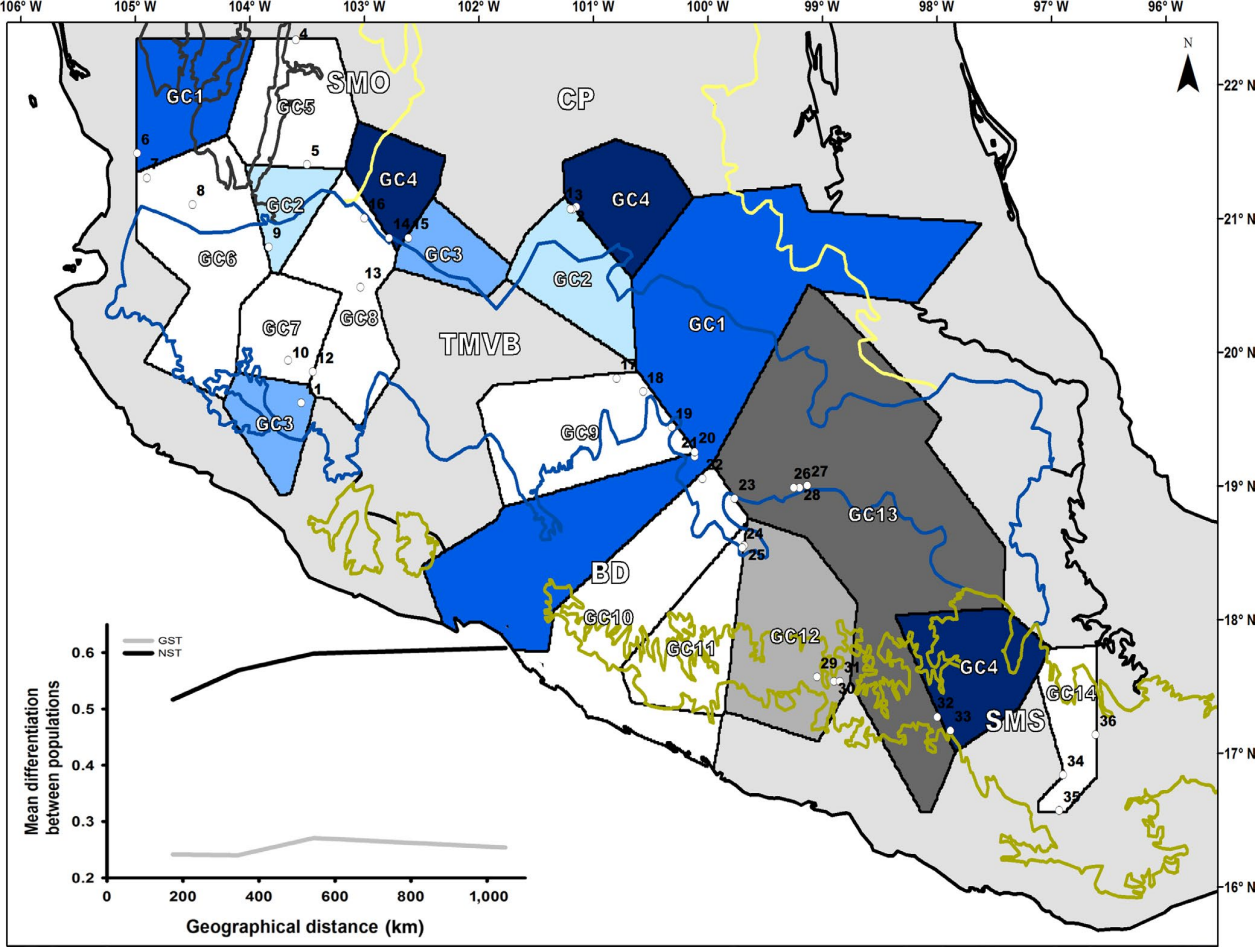
Geographical location of the most important genetic clusters for *Quercus castanea* using GENELAND. White polygons represent unique genetic clusters, blue scale polygons represent genetic clusters within the TMVB, and gray scale polygons represent genetic clusters formed by populations distributed in the TMVB and SMS. White circles represent *Q. castanea* studied populations. Each circle includes its population ID from Table 1. Bottom left corner includes the changes in the phylogeographic structure in terms of *G_ST_* and *N_ST_* per distance intervals

Globally, the population differentiation for ordered alleles (*N*_ST_) was 0.630 (0.089) and was 0.266 (0.022) for unordered alleles (*G*_ST_). The permutation test for the comparison of the two differentiation values was significant (*p* < .001), indicating phylogeographic structure in the populations of *Q. castanea*. When phylogeographic structure was analyzed considering the distance between oak populations, there were significant differences observed between *N*_ST_ and *G*_ST_ even at the lower distance intervals. However, it was also observed that phylogeographic structure expressed as *N*_ST_ > *G*_ST_ (*p* < .001) increased constantly as higher geographic distance intervals were considered (Figure 2).

Analysis of spatial genetic variation suggested the presence of 14 genetic clusters (*K* = 14) within *Q. castanea* after 10 independent runs (ln Pr(*K* = 14)= 1,152.97) (Figure 2). Genetic clusters number one and three (GC1, GC3) included noncontiguous populations distributed within the Trans‐Mexican Volcanic Belt. Genetic cluster number two (GC2) was formed by populations from the Central Plateau and the Trans‐Mexican Volcanic Belt. Genetic cluster number four (GC4) included dispersed populations through the Central Plateau, the Trans‐Mexican Volcanic Belt, and the Sierra Madre del Sur biogeographic regions. Several genetic clusters grouped contiguous populations through the Mexican Highlands biogeographic regions as follows: Sierra Madre Occidental (GC5), western Trans‐Mexican Volcanic Belt (GC6, GC7, and GC8), eastern Trans‐Mexican Volcanic Belt (GC9, GC10, and GC11), and Sierra Madre del Sur (GC14). Finally, genetic clusters 12 and 13 grouped noncontiguous populations that belong to the Trans‐Mexican Volcanic Belt and the Sierra Madre del Sur biogeographic regions.

### 3.3 Historical population demography

We obtained nonsignificant negative and positive *F_S_* values for all populations probably due to limited sampling sizes; therefore, mismatch distributions were not calculated at the population level (Table 3). However, when grouping samples at the regional level, a signal of recent demographic expansion was detected by both *F_S_* and Harpending's raggedness indexes for the Trans‐Mexican Volcanic Belt and the Sierra Madre del Sur (Table 3). Values of the time to expansion (*τ*) for both regions were 8.34 for the Sierra Madre del Sur and 8.49 for the Trans‐Mexican Volcanic Belt. This finding signifies that the demographic expansion could have occurred in the Sierra Madre del Sur between 5.9 Ma (low mutation rate, long generation time) and 151 ka (high mutation rate, short generation time). Similarly, the most recent expansion in the Trans‐Mexican Volcanic Belt would be dated between 6 Ma and 151 ka.

**Table 3:** Tests for historical population expansions and estimation of demographic parameters for 36 populations of *Quercus castanea* in four biogeographic provinces of Mexico according to Table 1 notation

Population ID	*F*_S_	Raggedness index	Ɵ_1_	Ɵ_2_	τ	−log[CL]
1	1.29	–	–	–	–	–
2	−1.53	–	–	–	–	–
3	1.35	–	–	–	–	–
**R1**	−1.18	–	–	–	–	–
4	−0.34	–	–	–	–	–
5	−0.38	–	–	–	–	–
**R2**	0.87	–	–	–	–	–
6	−0.97	–	–	–	–	–
7	−1.31	–	–	–	–	–
8	−1.18	–	–	–	–	–
9	0.27	–	–	–	–	–
10	1.66	–	–	–	–	–
11	0.68	–	–	–	–	–
12	0.01	–	–	–	–	–
13	−1.58	–	–	–	–	–
14	0.36	–	–	–	–	–
15	0.38	–	–	–	–	–
16	−0.67	–	–	–	–	–
17	−0.93	–	–	–	–	–
18	0.33	–	–	–	–	–
19	0.20	–	–	–	–	–
20	0.73	–	–	–	–	–
21	0.36	–	–	–	–	–
22	−0.41	–	–	–	–	–
23	0.10	–	–	–	–	–
24	2.93	–	–	–	–	–
25	0.59	–	–	–	–	–
26	−0.57	–	–	–	–	–
27	2.46	–	–	–	–	–
28	2.34	–	–	–	–	–
**R3**	**−25.00**	**0.01**	7.45E−01	3.88E + 01	8.34	231.73
29	1.99	–	–	–	–	–
30	−2.67	–	–	–	–	–
31	0.01	–	–	–	–	–
32	−0.04	–	–	–	–	–
33	0.49	–	–	–	–	–
34	1.21	–	–	–	–	–
35	0.64	–	–	–	–	–
36	1.45	–	–	–	–	–
**R4**	**−0.94**	**0.04**	2.21E−04	2.87E + 01	8.49	66.14

Note

Bold *F_S_* represent significant values; Θ_0_ and Θ_1_ are, respectively, ancestral and current population sizes scaled by mutation rate; τ is the number of generations since the expansion occurred, scaled by mutation rate; −log [CL] is the likelihood of the model. The absent values (–) represent noncalculated estimators due to the absence of *F_S_* significant values

### 3.4 Past and contemporary ecological niche modeling

**Figure 3:**
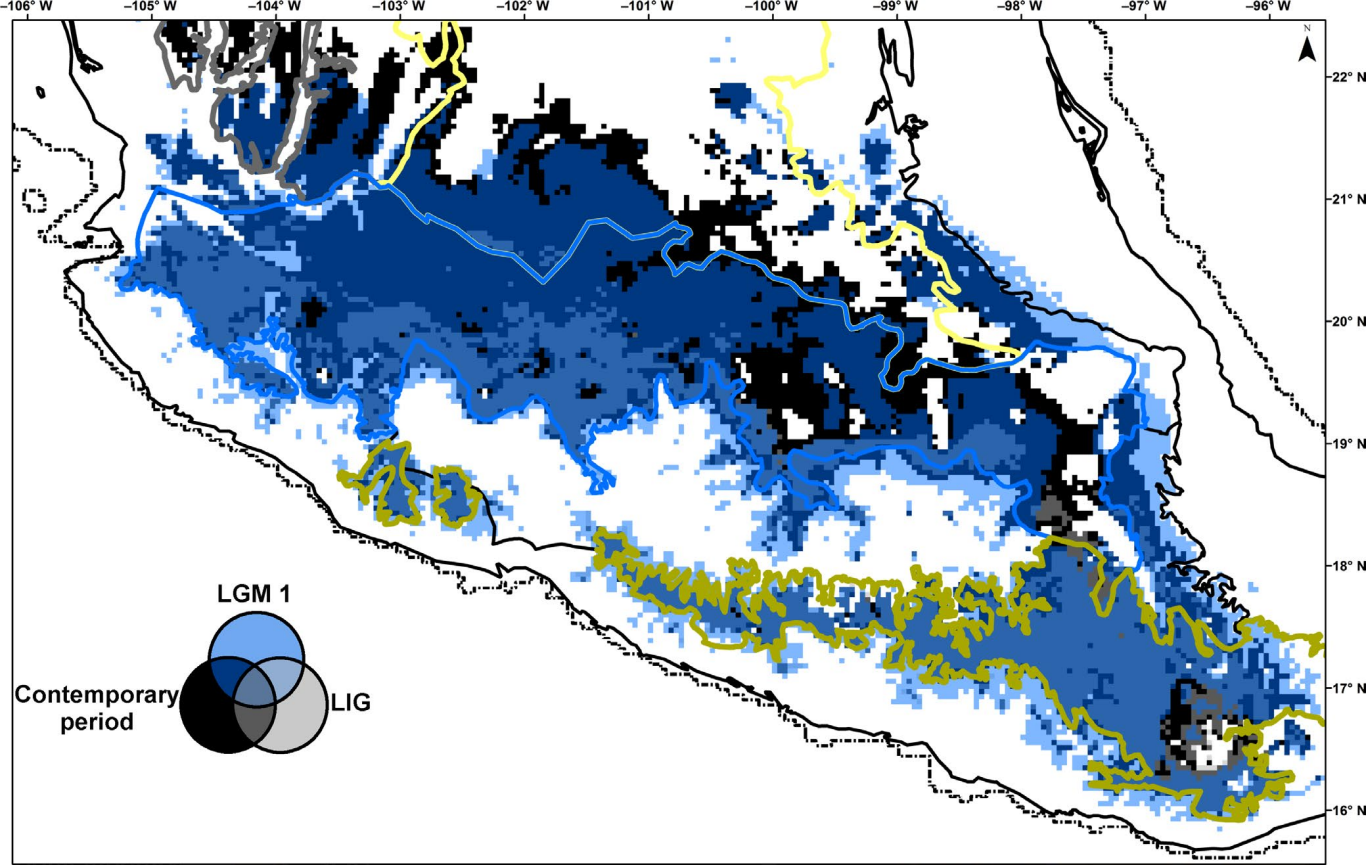
Compared *Quercus castanea* climatically suitable areas between the contemporary period, Last Glacial Maximum according to the Community Climate System Model CCSM (LGM1) and the Last interglacial (LIG). Dashed line corresponds to the expected Last Glacial Maximum shoreline. Colored lines represent the biogeographic regions of Mexico. Sierra Madre Occidental (gray), Central Plateu (yellow), Trans‐Mexican Volcanic Belt (blue), and Sierra Madre del Sur (green)

**Figure 4:**
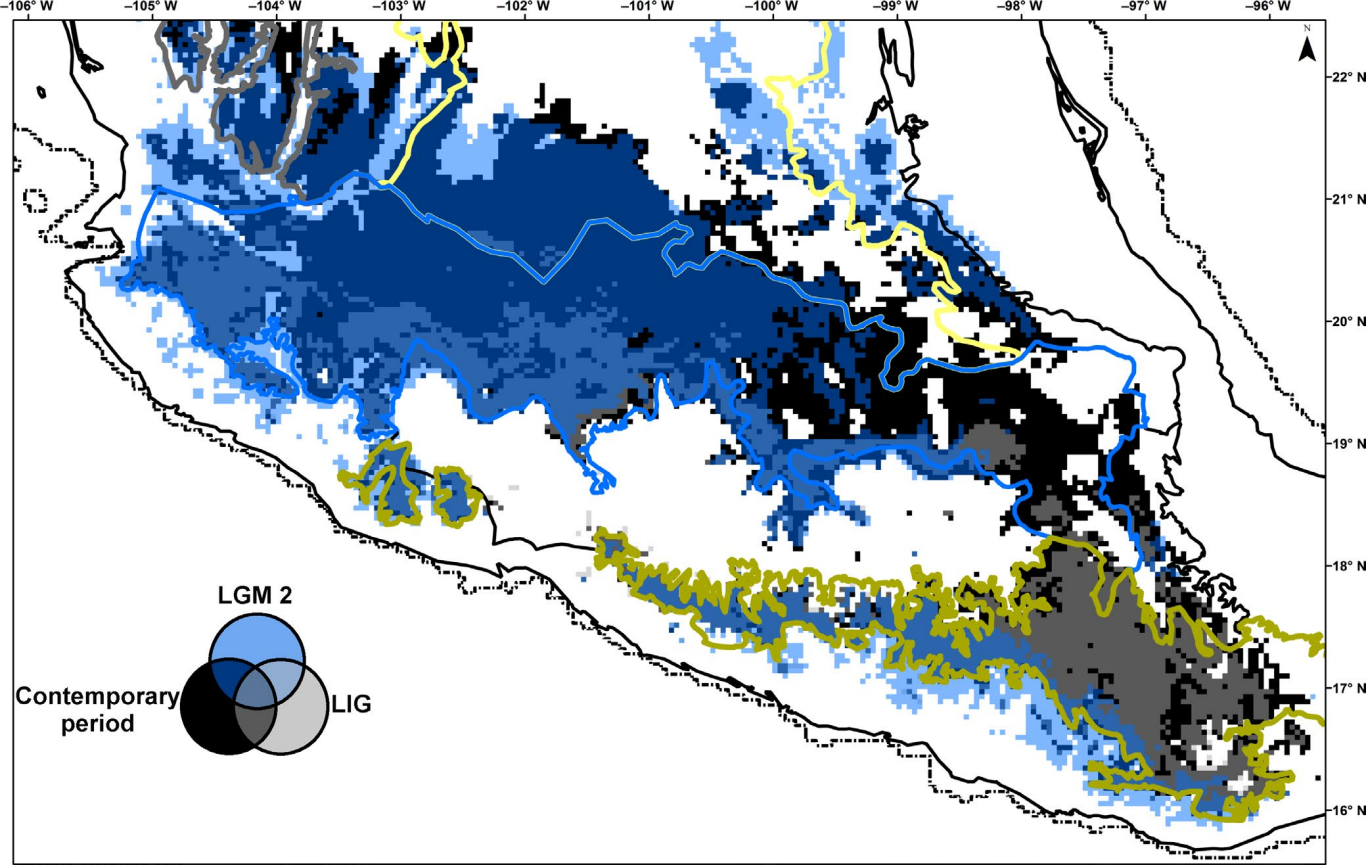
Compared *Quercus castanea* climatically suitable areas between the contemporary period, Last Glacial Maximum according to the Model for Interdisciplinary Research on Climate MIROC (LGM2) and the Last interglacial (LIG). Dashed line corresponds to the expected Last Glacial Maximum shoreline. Colored lines represent the biogeographic regions of Mexico. Sierra Madre Occidental (gray), Central Plateu (yellow), Trans‐Mexican Volcanic Belt (blue), and Sierra Madre del Sur (green)

The AUC values (averaged over the 100 runs) for the training and test data for the Maxent modeling were 0.971 and 0.963, indicating an appropriate model performance. The jackknife analysis indicated that the climatic variables with the highest relative contributions to the contemporary distribution model were related to elevation and temperature seasonality. The estimation of the distribution extent of *Q. castanea* suggests that 19% of the species suitable habitat has remained stable from the LIG to the contemporary period (Figures 3 and 4). Such stable habitat extends from the west to the east in the southern portion of the Trans‐Mexican Volcanic Belt and the western portion of the Sierra Madre del Sur. Models also suggested long‐term fragmentation and isolation of some areas. For example, all three models (LIG and two LGM) indicated that the suitable areas for *Q. castanea* in the Trans‐Mexican Volcanic Belt are not contiguous and show a reduction in extent from west to east. Similarly, the three models also coincided in indicating a discontinuity in the distribution of *Q. castanea* between the Sierra Madre del Sur and the Trans‐Mexican Volcanic Belt. The three models showed similar results for the Sierra Madre Occidental, the Central Plateau, western and central Trans‐Mexican Volcanic Belt, and western Sierra Madre del Sur. However, the two models of the LGM showed some differences in the distribution areas predicted in the eastern portions of the Trans‐Mexican Volcanic Belt and the Sierra Madre del Sur.

## 4 DISCUSSION

The analysis of the evolutionary patterns reported for the Mexican Highlands (e.g., Bryson, Linkem, & Pavón‐Vázquez, 2017; Bryson et al., 2018; Caviedes‐Solis & Leaché, 2018; Sanginés‐Franco et al., 2015; Villaseñor, Delgadillo, & Ortiz, 2006) have allowed to state hypothesis that describe the evolution of montane forests (e.g., cloud forest by Ramírez‐Barahona & Eguiarte, 2013), to describe common phylogeographical breaks limiting the species genetic structure and gene flow during different periods (Ornelas et al., 2013), and to propose the expected patterns of genetic variation in a heterogeneous geological framework mostly drove by volcanism (Mastretta‐Yanes et al., 2015). Such studies clearly illustrate the still incipient understanding of the biota evolution in a transition zone between the Nearctic and Neotropics. In this study, we present the results of the roles of tectonics and paleoclimatic factors could have shaped the genetic diversity and its distribution of a widely distributed species throughout Mexican mountains.

### 4.1 *Quercus castanea* genetic diversity

Interestingly, genetic variation that has been found in oak species using similar chloroplast molecular markers (cpSSR) is frequently high and has been explained as a product of complex interactions between geographical and environmental factors in several North American and Central American species, such as *Q. lobata* (Grivet, Duguilloux, Petit, & Sork, 2006; Grivet, Sork, Westfall, & Davis, 2008), *Q. crassipes*, *Q. crassifolia* (Tovar‐Sánchez, Mussali‐Galante, & Esteban‐Jiménez, 2008), *Q. affinis*, *Q. laurina* (Ramos‐Ortiz, Oyama, Rodríguez‐Correa, & González‐Rodríguez, 2016), *Q. deserticola* (Rodríguez‐Gómez et al., 2018), *Q. insignis*, *Q. sapotifolia* (Rodríguez‐Correa et al., 2017) and *Q. costarricensis* and *Q. bumeloides* (Rodríguez‐Correa et al., 2018). However, our results indicate that *Q. castanea* probably has the highest haplotype richness and intrapopulation genetic diversity compared with other oak species studied to date in the Americas. The only species we know of with higher haplotype diversity than *Q. castanea* are the evergreen Mediterranean oaks *Q. coccifera*, *Q. ilex,* and *Q. suber* (Jiménez, de Heredia, Collada, Lorenzo, & Gil, 2004; Lumaret, Mir, Michaud, & Raynal, 2008), for which introgression and the persistence of large populations through glacial cycles were proposed as the main causes for the observed diversity.

High levels of genetic diversity in natural populations of Mexican oaks may be attributed to processes such as introgression and hybridization (e.g., Albarrán‐Lara, Mendoza Cuenca, Valencia Ávalos, González Rodríguez, & Oyama, 2010; McCauley, Cortés‐Palomec, & Oyama, 2019; Oyama et al., 2018; Peñaloza‐Ramírez et al., 2010), which is important considering that hybridization could also occur between *Q. castanea* and other sympatric oak species (Valencia‐Cuevas, Piñero, Mussali‐Galante, Valencia‐Ávalos, & Tovar‐Sánchez, 2014). However, it is possible that in the case of *Q. castanea,* high genetic diversity is also related to the maintenance of large effective population sizes for a long period. Our estimations dated a population expansion possibly as old as 6 My ago, which coincides with the divergence time estimated for this species according to a recent phylogeny of the genus *Quercus* (Hipp et al., 2018). If after this expansion the species has maintained relatively large and constant population sizes and distribution range, there would have been considerable time for the accumulation of genetic variation. Ecological niche modeling suggested stable environmental habitat for the species since the late Pleistocence, the estimated extension of stable environmental conditions (representing 19% of the current climatic niche of the species) must have been enough for the maintenance of genetic variation. Therefore, it is likely that the most recent glacial and interglacial cycle of the Pleistocene had a minor effect on the distribution extent of *Q. castanea* populations allowing the species to constantly accumulate genetic variation.

Ecological niche modeling also indicated that, under the CCSM model, the areas suitable for *Q. castanea* in the Trans‐Mexican Volcanic Belt were connected with areas in the Central Plateau and the Sierra Madre del Sur, which may have promoted significant levels of gene flow and therefore the presence of shared haplotypes between biogeographic regions (Figure 3). In contrast, the MIROC model (with higher temperature and dryer conditions than the CCSM model) did not provide evidence of connectivity between the Trans‐Mexican Volcanic Belt and the Sierra Madre del Sur (Figure 4). Such difference between models, together with the pattern of haplotype sharing among biogeographic regions in *Q. castanea* populations, suggest that the moist forests model, as proposed by Ramírez‐Barahona and Eguiarte (2013) for the cloud forest, would be more appropriate for describing past population dynamics in this oak species. This model assumed the maintenance of genetic diversity throughout the species range, important levels of gene flow and population connectivity during the glacial periods.

From an evolutionary perspective, at the intraspecific level, *Q. castanea* could illustrate the rapid diversification of the genus *Quercus* in the Mexican highlands that has been related to ecological opportunity (Hipp et al., 2018) since this species currently has one of the widest ecological and geographical distributions, reflecting the species capacity to colonize and establish through a wide range of heterogeneous physiographic and climatic conditions.

### 4.2 *Quercus castanea* genetic structure

As has been reported for most of the oak species studied in the Americas (Cavender‐Bares et al., 2015; Cavender‐Bares, Gonzalez‐Rodriguez, Pahlich, Koehler, & Deacon, 2011; Gugger & Cavender‐Bares, 2013; Gugger, Ikegami, & Sork, 2013; McCauley et al., 2019; Rodríguez‐Gómez et al., 2018; Tovar‐Sánchez et al., 2008), *Q. castanea* also exhibited a well‐defined phylogeographic structure. However, unlike several of the mountainous oak and other tree species in Mexico and Central America (McCauley et al., 2019; Ornelas et al., 2013; Rodríguez‐Correa et al., 2017), the distribution of the genetic variation in this case is not explained by major biogeographic regions (Table 2; Figure 2) but by the subdivisions within the Trans‐Mexican Volcanic Belt and the Sierra Madre del Sur.

Within the Trans‐Mexican Volcanic Belt, genetic discontinuities in the western part were located between the Jalisco and the Sierra Madre Occidental blocks; GC1, GC2 and GC6 are at the Tepic‐Zacoalco rift (sensu Ferrari & Rosas‐Elguera, 2000), a Late Miocene to Quaternary tectonic depression that originated 5.5 My BP. Such populations differ significantly from each other and at the same time from populations located at the Sierra Madre Occidental (GC5) and at the southern portion of the Jalisco block (GC3, GC7, and GC8). Therefore, we propose that together, the extensional tectonics from the north to the northeast that produced the Tepic‐Zocoalco rift and the reduction in tectonic activity between 8 and 5 My BP (Ferrari & Rosas‐Elguera, 2000), could have affected the gene flow patterns of the species and therefore could explain the observed genetic differentiation between populations in this region.

Moving eastwards from the Tepic‐Zocoalco rift to the −101°W line, *Q. castanea* populations are genetically differentiated according to GENELAND results (Figure 2). Geologically, this region has different western and eastern crust dynamics (e.g., speed and position) and volcanism (Ferrari et al., 2012). Palynological records indicate drier environmental conditions in the eastern Trans‐Mexican Volcanic Belt compared to its western portion during the Pleistocene (Bradbury, 1997; del Socorro Lozano‐García & Ortega‐Guerrero, 1998). Together, geological and paleoclimatic differences at both sides of the Trans‐Mexican Volcanic Belt may have been important drivers of the genetic isolation between nearby populations. Similarly, in the Sierra Madre del Sur the differentiation among nearby populations was also observed. In that sense, the distribution of genetic clusters coincides with the region's diverse physiography as follows: the Cordillera Costera del Sur (GC 12), the Mixteca Alta (GC 4), the Sierras de Oaxaca and Sierras Orientales (GC 14), of which the last two are also associated with lithospheric blocks in southern Mexico, such as the Oaxaca and Juárez terrane (Cerca, Ferrari, López‐Martínez, Martiny, & Iriondo, 2007).

Interestingly, haplotypes located in the eastern Trans‐Mexican Volcanic Belt are frequently related to Sierra Madre del Sur haplotypes. Such results may be due to continuous historical connections from the eastern Trans‐Mexican Volcanic Belt and the Sierra Madre del Sur (Corona, Toledo, & Morrone, 2007; Ortíz‐Medrano, Moreno‐Letelier, & Piñero, 2008; Ruiz‐Sanchez, Rodriguez‐Gomez, & Sosa, 2012; Salas‐Lizana, Santini, Miranda‐Pérez, & Piñero, 2012) favored by population expansions. Such processes that presumably resulted in periods of high dispersal and contact between the pine–oak forests from the Trans‐Mexican Volcanic Belt and the Sierra Madre del Sur during the Pleistocene, have been reported for temperate forests by authors such as del Socorro Lozano‐García and Ortega‐Guerrero (1998), whom proposed that pine–oak forests have expanded downwards forming large continuous stands to later experienced upward contractions (Metcalfe, 2006).

### 4.3 *Quercus castanea* evolution in the Mexican highlands

Recently, authors such as Mastretta‐Yanes et al. (2015) and Caballero‐Rodríguez et al. (2018) have highlighted the role of geology and climate as major drivers of the distribution of the biota in the Mexican highlands since the Pliocene. Mastretta‐Yanes et al. (2015) proposed several scenarios that highlighted the expected effects of the interaction of climate and volcanism on the biological communities in the Mexican highlands. Such interactions seem to explain our findings as *Q. castanea* exhibited a complex genetic structure associated with physiographic regions with high volcanic activity and evidence of gene flow among biogeographic regions during glacial cycles.

Besides the evidence that geological events predating the early Pleistocene and a complex physiographic landscape explain the phylogeographic structure observed in *Q. castanea,* it is interesting that the historical demography analyses also suggested ancient signatures of demographic expansion (as old as ~ 6 My for both the Trans‐Mexican Volcanic Belt and the Sierra Madre del Sur). Older dates for the demographic expansion seem more credible than younger ones considering the expected long periods needed to allow the accumulation of cpDNA genetic variation. The ENM analyses also support the idea of a stable enough distribution for *Q. castanea* to maintain large populations*,* at least since the LIG with moderate range expansions and contractions during the last glacial cycle. Therefore, it is plausible that in response to climate variation, populations of this species mostly experienced altitudinal displacements and a series of expansions and contractions rather than large latitudinal migrations, favoring continuous levels of gene flow and promoting a species distribution rearrangement as it has been proposed for Neotropical species (Bennett, Bhagwat, & Willis, 2012).

Finally, our results indicate a similar pattern to previous studies in the Mexican highlands that have reported pre‐Quaternary divergence times (Bryson et al., 2017, 2018; Bryson & Riddle, 2012; Ornelas & González, 2014) and to patterns of pre‐Quaternary diversification for Neotropical species, characterized by complex mixtures of taxa that radiated and spread over a wide range of timescales (Bennett et al., 2012). These findings indicate that the origin and maintenance of a large proportion of the genetic diversity in *Q. castanea* have occurred within the Trans‐Mexican Volcanic Belt, which is also a hotspot for oak species diversity and has acted as a natural bridge between other biogeographic provinces (Bryson et al., 2018).

## 5 CONCLUSIONS

Our study suggests that together tectonics and paleoclimate have shaped the genetic structure of *Q. castanea*, as has been found for other species in the Mexican highlands (Mastretta‐Yanes et al., 2015). By focusing on species with a wide distribution, we were able to reveal in detail aspects of historical population dynamics such as old population expansion predating the Pleistocene and the maintenance of considerable areas that probably sustained stable populations over the Pleistocene climatic changes. The presence of these stable areas favored the genetic connectivity between biogeographic provinces (mostly between the TMVB and the SMS) and the maintenance of genetic variation. These results are congruent with the “moist forests model” proposed by Ramírez‐Barahona and Eguiarte (2013) for the Pleistocene dynamics of cloud forests indicating that oak species in Mexico and Central America could be useful models to test evolutionary hypotheses in Neotropical environments.

## CONFLICT OF INTEREST

Authors have no competing interests to declare.

## AUTHOR CONTRIBUTIONS

JMPR and KO conceived and designed the study. JMPR and VRR performed field and laboratory work. JMPR, HRC, AGR, and KO analyzed and interpreted the data. JMPR, HRC, and KO drafted the article. All authors contributed equally to subsequent drafts and gave final approval for publication.

## Data Availability

Genetic and geographic data are available through xlsx documents in the Dryad repository at https://doi.org/10.5061/dryad.g4f4qrfm4.

## References

[ece36189-bib-0001] Albarrán Lara, A. , Mendoza Cuenca, L. , Valencia Ávalos, S. , González Rodríguez, A. , & Oyama, K. (2010). Leaf fluctuating asymmetry increases with hybridization and introgression between *Quercus magnoliifolia* and *Quercus resinosa* (Fagaceae) through an altitudinal gradient in Mexico. International Journal of Plant Sciences, 171, 310–322. 10.1086/650317

[ece36189-bib-0002] Amante, C. , & Eakins, B. W. (2009). ETOPO1 1 Arc‐minute global relief model: Procedures, data sources and analysis. NOAA Technical Memorandum NESDIS, NGDC‐24, 19 pp.

[ece36189-bib-0003] Ashley, M. V. , Backs, J. R. , Kindsvater, L. , & Abraham, S. T. (2018). Genetic variation and structure in an endemic island oak, *Quercus tomentella*, and mainland canyon oak, *Quercus chrysolepis* . International Journal of Plant Sciences, 179, 151–161. 10.1086/696023

[ece36189-bib-0004] Bandelt, H.‐J.‐J. , Forster, P. , Sykes, B. C. , & Richards, M. B. (1995). Mitochondrial portraits of human populations using median networks. Genetics, 141, 743–753. 8647407 10.1093/genetics/141.2.743 PMC1206770

[ece36189-bib-0005] Bennett, K. , Bhagwat, S. , & Willis, K. (2012). Neotropical refugia. The Holocene, 22, 1207–1214. 10.1177/0959683612450204

[ece36189-bib-0006] Bradbury, J. P. (1997). Sources of glacial moisture in Mesoamerica. Quat Int, 43–44, 97–110. 10.1016/S1040-6182(97)00025-6

[ece36189-bib-0007] Bryson, R. W. , Linkem, C.W. , Pavón‐Vázquez, C.J. , Nieto‐Montes de Oca, A. , Klicka, J. , & McCormack, J.E. (2017). A phylogenomic perspective on the biogeography of skinks in the Plestiodon brevirostris group inferred from target enrichment of ultraconserved elements. Journal of Biogeography, 44, 2033–2044. 10.1111/jbi.12989

[ece36189-bib-0008] Bryson, R. W. , & Riddle, B. R. (2012). Tracing the origins of widespread highland species: A case of Neogene diversification across the Mexican sierras in an endemic lizard. Biological Journal of the Linnean Society, 105, 382–394. 10.1111/j.1095-8312.2011.01798.x

[ece36189-bib-0009] Bryson, R. W. , Zarza, E. , Grummer, J. A. , Parra‐Olea, G. , Flores‐Villela, O. , Klicka, J. , & McCormack, J. E. (2018). Phylogenomic insights into the diversification of salamanders in the *Isthmura bellii* group across the Mexican highlands. Molecular Phylogenetics and Evolution, 125, 78–84. 10.1016/j.ympev.2018.03.024 29555294

[ece36189-bib-0010] Caballero, M. , Lozano‐García, S. , Ortega‐Guerrero, B. , & Correa‐Metrio, A. (2019). Quantitative estimates of orbital and millennial scale climatic variability in central Mexico during the last 40,000 years. Quaternary Science Reviews, 205, 62–75. 10.1016/J.QUASCIREV.2018.12.002

[ece36189-bib-0011] Caballero‐Rodríguez, D. , Correa‐Metrio, A. , Lozano‐García, S. , Sosa‐Nájera, S. , Ortega, B. , Sanchez‐Dzib, Y. , … Sandoval‐Montaño, A. (2018). Late‐Quaternary spatiotemporal dynamics of vegetation in Central Mexico. Review of Palaeobotany and Palynology, 250, 44–52. 10.1016/J.REVPALBO.2017.12.004

[ece36189-bib-0012] Cavender‐Bares, J. (2019). Diversification, adaptation, and community assembly of the American oaks (*Quercus*), a model clade for integrating ecology and evolution. New Phytologist, 221, 669–692. 10.1111/nph.15450 30368821

[ece36189-bib-0013] Cavender‐Bares, J. , González‐Rodríguez, A. , Eaton, D. A. R. , Hipp, A. A. , Beulke, A. , & Manos, P. S. (2015). Phylogeny and biogeography of the american live oaks (*Quercus* subsection Virentes): A genomic and population genetics approach. Molecular Ecology, 24, 3668–3687. 10.1111/mec.13269 26095958

[ece36189-bib-0014] Cavender‐Bares, J. , Gonzalez‐Rodriguez, A. , Pahlich, A. , Koehler, K. , & Deacon, N. (2011). Phylogeography and climatic niche evolution in live oaks (*Quercus* series Virentes) from the tropics to the temperate zone. Journal of Biogeography, 38, 962–981. 10.1111/j.1365-2699.2010.02451.x

[ece36189-bib-0015] Caviedes‐Solis, I. W. , & Leaché, A. D. (2018). Leapfrogging the Mexican highlands: Influence of biogeographical and ecological factors on the diversification of highland species. Biological Journal of the Linnean Society, 123, 767–781. 10.1093/biolinnean/bly002

[ece36189-bib-0016] Cerca, M. , Ferrari, L. , López‐Martínez, M. , Martiny, B. , & Iriondo, A. (2007). Late Cretaceous shortening and early Tertiary shearing in the central Sierra Madre del Sur, southern Mexico: Insights into the evolution of the Caribbean‐North American plate interaction. Tectonics, 26,,1–34. 10.1029/2006TC001981

[ece36189-bib-0017] Collins, W. D. , Bitz, C. M. , Blackmon, M. L. , Bonan, G. B. , Bretherton, C. S. , Carton, J. A. , … Smith, R. D. (2006). The community climate system model Version 3 (CCSM3). Journal of Climate, 19, 2122–2143. 10.1175/JCLI3761.1

[ece36189-bib-0018] Corona, A. M. , Toledo, V. H. , & Morrone, J. J. (2007). Does the Trans‐Mexican Volcanic Belt represent a natural biogeographical unit? An analysis of the distributional patterns of Coleoptera. Journal of Biogeography, 34, 1008–1015. 10.1111/j.1365-2699.2006.01666.x

[ece36189-bib-0019] De Cserna, Z. (1989). An outline of the geology of Mexico In Bally A. W., & Plamer A. R. (Eds.), The Geology of North America–An Overview (pp. 233–264). Boulder, CO: Geological Society of America.

[ece36189-bib-0020] Deguilloux, M.‐F. , Dumolin‐Lapègue, S. , Gielly, L. , et al (2003). A set of primers for the amplification of chloroplast microsatellites in *Quercus* . Molecular Ecology Notes, 3, 24–27. 10.1046/j.1471-8286.2003.00339.x

[ece36189-bib-0021] del Socorro Lozano‐García, M. , & Ortega‐Guerrero, B. (1998). Late Quaternary environmental changes of the central part of the Basin of Mexico; correlation between Texcoco and Chalco basins. Review of Palaeobotany and Palynology, 99, 77–93. 10.1016/S0034-6667(97)00046-8

[ece36189-bib-0022] Excoffier, L. , Laval, G. , & Schneider, S. (2007). Arlequin (version 3.0): An integrated software package for population genetics data analysis. Evol Bioinform Online, 1, 47–50. 19325852 PMC2658868

[ece36189-bib-0023] Ferrari, L. , Orozco‐Esquivel, T. , Manea, V. , & Manea, M. (2012). The dynamic history of the Trans‐Mexican Volcanic Belt and the Mexico subduction zone. Tectonophysics, 522–523, 122–149. 10.1016/J.TECTO.2011.09.018

[ece36189-bib-0024] Ferrari, L. , & Rosas‐Elguera, J. (2000). Late Miocene to Quaternary extension at the northern boundary of the Jalisco block, western Mexico: The Tepic‐Zaocalco rift revisited. Spec Pap Geol Soc Am, 334, 41–63. 10.1130/0-8137-2334-5.41

[ece36189-bib-0025] Ferrusquía‐Villafranca, I. (1993). Geology of Mexico: A synopsis In Ramamoorthy T. P., Bye R., Lot A., & Fa J. (Eds.), Biological diversity of Mexico: Origins and distribution (pp. 3–107). New York, NY: Oxford University Press.

[ece36189-bib-0026] Fielding, A. H. , & Bell, J. F. (1997). A review of methods for the assessment of prediction errors in conservation presence/absence models. Environmental Conservation, 24, 38–49. 10.2307/44519240

[ece36189-bib-0027] Fu, Y.‐X. (1997). Statistical tests of neutrality of mutations against population growth, hitchhiking and background selection. Genetics, 147, 915–925. 9335623 10.1093/genetics/147.2.915 PMC1208208

[ece36189-bib-0028] Goldstein, D. B. , Ruiz Bares, A. , Cavalli‐Sforzaf, L. L. , & Feldman, M. W. (1995). An evaluation of genetic distances for use with microsatellite loci. Genetics, 139, 463–471. 7705647 10.1093/genetics/139.1.463 PMC1206344

[ece36189-bib-0029] Grivet, D. , Duguilloux, M.‐F. , Petit, R. J. , & Sork, V. L. (2006). Contrasting patterns of historical colonization in white oaks (*Quercus* spp.) in California and Europe. Molecular Ecology, 15, 4085–4093. 10.1111/j.1365-294X.2006.03083.x 17054504

[ece36189-bib-0030] Grivet, D. , Sork, V. L. , Westfall, R. D. , & Davis, F. W. (2008). Conserving the evolutionary potential of California valley oak (*Quercus lobata* Née): A multivariate genetic approach to conservation planning. Molecular Ecology, 17, 139–156. 10.1111/j.1365-294X.2007.03498.x 17868293

[ece36189-bib-0031] Gugger, P. F. , & Cavender‐Bares, J. (2013). Molecular and morphological support for a Florida origin of the Cuban oak. Journal of Biogeography, 40, 632–645. 10.1111/j.1365-2699.2011.02610.x

[ece36189-bib-0032] Gugger, P. F. , Ikegami, M. , & Sork, V. L. (2013). Influence of late Quaternary climate change on present patterns of genetic variation in valley oak, *Quercus lobata* Née. Molecular Ecology, 22, 3598–3612. 10.1111/mec.12317 23802553

[ece36189-bib-0033] Guillot, G. , Renaud, S. , Ledevin, R. , Michaux, J. , & Claude, J. (2012). A unifying model for the analysis of phenotypic, genetic, and geographic data. Systematic Biology, 61, 897–911. 10.1093/sysbio/sys038 22398122

[ece36189-bib-0034] Gutiérrez‐García, T. A. , & Vázquez‐Domínguez, E. (2013). Consensus between genes and stones in the biogeographic and evolutionary history of Central America. Quat Res, 79, 311–324. 10.1016/j.yqres.2012.12.007

[ece36189-bib-0035] Hardy, O. J. , & Vekemans, X. (2002). spagedi: A versatile computer program to analyse spatial genetic structure at the individual or population levels. Molecular Ecology Notes, 2, 618–620. 10.1046/j.1471-8286.2002.00305.x

[ece36189-bib-0036] Harpending, H. C. (1994). Signature of ancient population growth in a low‐resolution mitochondrial DNA mismatch distribution. Human Biology, 66, 591–600. 8088750

[ece36189-bib-0037] Hasumi, H. , & Emori, S. (2004). K‐1 coupled GCM (MIROC) description. Tokyo, Japan: Center for Climate System Research, University of Tokyo.

[ece36189-bib-0038] Herrera‐Arroyo, M. L. , Sork, V. L. , González‐Rodríguez, A. , Rocha‐Ramírez, V. , Vega, E. , & Oyama, K. (2013). Seed‐mediated connectivity among fragmented populations of *Quercus castanea* (Fagaceae) in a Mexican landscape. American Journal of Botany, 100, 1663–1671. 10.3732/ajb.1200396 23942083

[ece36189-bib-0039] Heuertz, M. , Teufel, J. , González‐Martínez, S. C. , et al (2010). Geography determines genetic relationships between species of mountain pine (*Pinus mugo* complex) in western Europe. Journal of Biogeography, 37, 541–556. 10.1111/j.1365-2699.2009.02223.x

[ece36189-bib-0040] Hijmans, R. J. , Cameron, S. E. , Parra, J. L. , Jones, P. G. , & Jarvis, A. (2005). Very high resolution interpolated climate surfaces for global land areas. International Journal of Climatology, 25, 1965–1978. 10.1002/joc.1276

[ece36189-bib-0041] Hipp, A. L. , Eaton, D. A. R. , Cavender‐Bares, J. , Fitzek, E. , Nipper, R. , & Manos, P. S. (2014). A framework phylogeny of the American oak clade based on sequenced RAD data. PLoS ONE, 9, e93975 10.1371/journal.pone.0093975 24705617 PMC3976371

[ece36189-bib-0042] Hipp, A. L. , Manos, P. S. , González‐Rodríguez, A. , Hahn, M. , Kaproth, M. , McVay, J. D. , … Cavender‐Bares, J. (2018). Sympatric parallel diversification of major oak clades in the Americas and the origins of Mexican species diversity. New Phytologist, 217, 439–452. 10.1111/nph.14773 28921530

[ece36189-bib-0043] Hipp, A. L. , Manos, P. S. , Hahn, M. , Avishai, M. , Bodénès, C. , Cavender‐Bares, J. , … Valencia‐Avalos, S. (2019). Genomic landscape of the global oak phylogeny. New Phytologist, , 1–15. 10.1111/nph.16162 31609470

[ece36189-bib-0044] Jiménez, P. , de Heredia, U. L. , Collada, C. , Lorenzo, Z. , & Gil, L. (2004). High variability of chloroplast DNA in three Mediterranean evergreen oaks indicates complex evolutionary history. Heredity, 93, 510–515. 10.1038/sj.hdy.6800551 15329661

[ece36189-bib-0045] Kappelle, M. (2006). Ecology and Conservation of Neotropical Montane Oak Forests. Ecological Studies Series, Vol. 185. Berlin – Heidelberg – New York: Springer Verlag, 483 pp.

[ece36189-bib-0046] Lefort, F. , & Douglas, G. C. (1999). An efficient micro‐method of DNA isolation from mature leaves of four hardwood tree species *Acer*, *Fraxinus*, *Prunus* and *Quercus* . Annals of Forest Science, 56, 259–263. 10.1051/forest:19990308

[ece36189-bib-0047] Lumaret, R. , Mir, C. , Michaud, H. , & Raynal, V. (2008). Phylogeographical variation of chloroplast DNA in holm oak (*Quercus ilex* L.). Molecular Ecology, 11, 2327–2336. 10.1046/j.1365-294X.2002.01611.x 12406243

[ece36189-bib-0048] Magni, C. R. , Ducousso, A. , Caron, H. , Petit, R. J. , & Kremer, A. (2005). Chloroplast DNA variation of *Quercus rubra* L. in North America and comparison with other Fagaceae. Molecular Ecology, 14, 513–524. 10.1111/j.1365-294X.2005.02400.x 15660942

[ece36189-bib-0049] Marsico, T. D. , Hellmann, J. J. , & Romero‐Severson, J. (2009). Patterns of seed dispersal and pollen flow in *Quercus garryana* (Fagaceae) following post‐glacial climatic changes. Journal of Biogeography, 36, 929–941. 10.1111/j.1365-2699.2008.02049.x

[ece36189-bib-0050] Mastretta‐Yanes, A. , Moreno‐Letelier, A. , Piñero, D. , Jorgensen, T. H. , & Emerson, B. C. (2015). Biodiversity in the Mexican highlands and the interaction of geology, geography and climate within the Trans‐Mexican Volcanic Belt. Journal of Biogeography, 42, 1586–1600. 10.1111/jbi.12546

[ece36189-bib-0051] McCauley, R. , Cortés‐Palomec, A. , & Oyama, K. (2019). Species diversification in a lineage of Mexican red oak (*Quercus* section *Lobatae* subsection *Racemiflorae*) – the interplay between distance, habitat and hybridization. Tree Genetics and Genomes, 15, 27 10.1007/s11295-019-1333-x

[ece36189-bib-0052] Meehl, G. A. , & Bony, S. (2011). Introduction to CMIP5. CLIVAR Exch, 56, 4–5.

[ece36189-bib-0053] Metcalfe, S. E. (2006). Late Quaternary environments of the northern deserts and central Transvolcanic Belt of Mexico. Ann Missouri Bot Gard, 93, 258–273. 0026-6493(2006)93%5B258:LQEOTN%5D2.0.CO;2

[ece36189-bib-0054] Morrone, J. J. (2005). Hacia una síntesis biogeográfica de México. Rev Mex Biodivers, 76, 207–252.

[ece36189-bib-0055] Morrone, J. J. (2010). Fundamental biogeographic patterns across the Mexican Transition Zone: An evolutionary approach. Ecography, 33, 355–661. 10.1111/j.1600-0587.2010.06266.x

[ece36189-bib-0056] Morrone, J. J. (2014). Biogeographical regionalisation of the neotropical region, Vol. 3782. 10.11646/zootaxa.3782.1.1 24871951

[ece36189-bib-0057] Navascués, M. , & Emerson, B. C. (2005). Chloroplast microsatellites: Measures of genetic diversity and the effect of homoplasy. Molecular Ecology, 14, 1333–1341. 10.1111/j.1365-294X.2005.02504.x 15813774

[ece36189-bib-0058] Navascués, M. , Hardy, O. J. , & Burgarella, C. (2009). Characterization of demographic expansions from pairwise comparisons of linked microsatellite haplotypes. Genetics, 181, 1013–1019. 10.1534/genetics.108.098194 19104073 PMC2651038

[ece36189-bib-0059] Nogués‐Bravo, D. (2009). Predicting the past distribution of species climatic niches. Global Ecology and Biogeography, 18, 521–531. 10.1111/j.1466-8238.2009.00476.x

[ece36189-bib-0060] Ornelas, J. F. , & González, C. (2014). Interglacial genetic diversification of *Moussonia deppeana* (Gesneriaceae), a hummingbird‐pollinated, cloud forest shrub in northern Mesoamerica. Molecular Ecology, 23, 4119–4136. 10.1111/mec.12841 24954419

[ece36189-bib-0061] Ornelas, J. F. , Sosa, V. , Soltis, D. E. , Daza, J. M. , González, C. , Soltis, P. S. , … Ruiz‐Sanchez, E. (2013). Comparative phylogeographic analyses illustrate the complex evolutionary history of threatened cloud forests of northern Mesoamerica. PLoS ONE, 8, e56283 10.1371/journal.pone.0056283 23409165 PMC3567015

[ece36189-bib-0062] Ortíz‐Medrano, A. , Moreno‐Letelier, A. , & Piñero, D. (2008). Fragmentación y expansión demográfica en las poblaciones mexicanas de *Pinus ayacahuite* var. *ayacahuite* . Bol La Soc Botánica México, 83, 25–36.

[ece36189-bib-0063] Otto‐Bliesner, B. L. , Marshall, S. J. , Overpeck, J. T. , Miller, G. H. , & Hu, A. ,. CAPE Last Interglacial Project members (2008). Simulating Arctic climate warmth and icefield retreat in the last interglaciation. Nature, 311, 1751–1753. 10.1126/science.1120808 16556838

[ece36189-bib-0064] Oyama, K. , Herrera‐Arroyo, M. L. , Rocha‐Ramírez, V. , Benítez‐Malvido, J. , Ruiz‐Sánchez, E. , & González‐Rodríguez, A. (2017). Gene flow interruption in a recently human‐modified landscape: The value of isolated trees for the maintenance of genetic diversity in a Mexican endemic red oak. Forest Ecology and Management, 390, 27–35. 10.1016/j.foreco.2017.01.018

[ece36189-bib-0065] Oyama, K. , Ramírez‐Toro, W. , Peñaloza‐Ramírez, J. M. , Pérez‐Pedraza, A. E. , Torres‐Miranda, C. A. , Ruiz‐Sánchez, E. , & González‐Rodríguez, A. (2018). High genetic diversity and connectivity among populations of *Quercus candicans*, *Q. crassifolia* and *Q. castanea* in a heterogeneous landscape in Mexico. Trop Conserv Sci, 11, 1–14. 10.1177/1940082918766195

[ece36189-bib-0066] Peñaloza Ramírez, J. , González Rodríguez, A. , Mendoza Cuenca, L. , Henri, C. , Kremer, A. , & Oyama, K. (2010). Interspecific gene flow in a multispecies oak hybrid zone in the Sierra Tarahumara of Mexico. Annals of Botany, 105, 389–399. 10.1093/aob/mcp303 20056653 PMC2826251

[ece36189-bib-0067] Phillips, S. J. , Anderson, R. P. , & Schapire, R. E. (2006). Maximum entropy modeling of species geographic distributions. Ecol Modell, 190, 231–259. 10.1016/J.ECOLMODEL.2005.03.026

[ece36189-bib-0068] Polzin, T. , & Daneshmand, S. V. (2003). On Steiner trees and minimum spanning trees in hypergraphs. Operations Research Letters, 31, 12–20. 10.1016/S0167-6377(02)00185-2

[ece36189-bib-0069] Pons, O. , & Petitt, R. J. (1996). Measuring and testing genetic differentiation with ordered versus unordered alleles. Genetics, 144, 1237–1245. 8913764 10.1093/genetics/144.3.1237 PMC1207615

[ece36189-bib-0070] R Core Team (2017). R: A language and environment for statistical computing. Vienna, Austria: R Foundation for Statistical Computing https://www.R-project.org/

[ece36189-bib-0071] Ramírez‐Barahona, S. , & Eguiarte, L. E. (2013). The role of glacial cycles in promoting genetic diversity in the Neotropics: The case of cloud forests during the Last Glacial Maximum. Ecology and Evolution, 3, 725–738. 10.1002/ece3.483 23531632 PMC3605859

[ece36189-bib-0072] Ramos‐Onsins, S. E. , & Rozas, J. (2002). Statistical properties of new neutrality tests against population growth. Molecular Biology and Evolution, 19, 2092–2100. 10.1093/oxfordjournals.molbev.a004034 12446801

[ece36189-bib-0073] Ramos‐Ortiz, S. , Oyama, K. , Rodríguez‐Correa, H. , & González‐Rodríguez, A. (2016). Geographic structure of genetic and phenotypic variation in the hybrid zone between *Quercus affinis* and *Q. laurina* in Mexico. Plant Species Biology, 31, 219–232. 10.1111/1442-1984.12109

[ece36189-bib-0074] Ríos‐Muñoz, C. A. , & Navarro‐Sigüenza, A. G. (2012). Patterns of species richness and biogeographic regionalization of the avifaunas of the seasonally dry tropical forest in Mesoamerica. Studies on Neotropical Fauna and Environment, 47(3), 171–182. 10.1080/01650521.2012.734175

[ece36189-bib-0075] Rodríguez‐Correa, H. , Oyama, K. , Quesada, M. , Fuchs, E. J. , & González‐Rodríguez, A. (2018). Contrasting patterns of population history and seed‐mediated gene flow in two endemic Costa Rican Oak species. Journal of Heredity, 109, 530–542. 10.1093/jhered/esy011 29509902

[ece36189-bib-0076] Rodríguez‐Correa, H. , Oyama, K. , Quesada, M. , Fuchs, E. J. , Quezada, M. , Ferrufino, L. , … González‐Rodríguez, A. (2017). Complex phylogeographic patterns indicate Central American origin of two widespread Mesoamerican *Quercus* (Fagaceae) species. Tree Genetics and Genomes, 13, 62 10.1007/s11295-017-1147-7

[ece36189-bib-0077] Rodríguez‐Gómez, F. , Oyama, K. , Ochoa‐Orozco, M. , Mendoza‐Cuenca, L. , Gaytán‐Legaria, R. , & González‐Rodríguez, A. (2018). Phylogeography and climate‐associated morphological variation in the endemic white oak *Quercus deserticola* (Fagaceae) along the Trans‐Mexican Volcanic Belt. Botany‐Botanique, 96, 121–131. 10.1139/cjb-2017-0116

[ece36189-bib-0078] Rogers, A. R. , & Harpending, H. (1992). Population growth makes waves in the distribution of pairwise genetic differences. Molecular Biology and Evolution, 9, 552–569. 10.1093/oxfordjournals.molbev.a040727 1316531

[ece36189-bib-0079] Ruiz‐Sanchez, E. , Rodriguez‐Gomez, F. , & Sosa, V. (2012). Refugia and geographic barriers of populations of the desert poppy, *Hunnemannia fumariifolia* (Papaveraceae). Organisms, Diversity, and Evolution, 12, 133–143. 10.1007/s13127-012-0089-z

[ece36189-bib-0080] Rzedowski, J. (1978). Vegetación de México. México: CONABIO, Editorial Limusa.

[ece36189-bib-0081] Safner, T. , Miller, M. P. , McRae, B. H. , Fortin, M.‐J. , & Manel, S. (2011). Comparison of Bayesian clustering and edge detection methods for inferring boundaries in landscape genetics. International Journal of Molecular Sciences, 12, 865–889. 10.3390/ijms12020865 21541031 PMC3083678

[ece36189-bib-0082] Salas‐Lizana, R. , Santini, N. S. , Miranda‐Pérez, A. , & Piñero, D. I. (2012). The Pleistocene glacial cycles shaped the historical demography and phylogeography of a pine fungal endophyte. Mycological Progress, 11, 569–581. 10.1007/s11557-011-0774-x

[ece36189-bib-0083] Sanginés‐Franco, C. , Luna‐Vega, I. , Contreras‐Medina, R. , Espinosa, D. , Tejero‐Díez, J. D. , & Rivas, G. (2015). Diversity, endemism and conservation of ferns (Polypodiales) in the Mexican mountain component. Journal of Mountain Science, 12, 891–904. 10.1007/s11629-014-3070-9

[ece36189-bib-0084] Sebastiani, F. , Carnevale, S. , & Vendramin, G. G. (2004). A new set of mono‐ and dinucleotide chloroplast microsatellites in Fagaceae. Molecular Ecology Notes, 4, 259–261. 10.1111/j.1471-8286.2004.00635.x

[ece36189-bib-0085] Sosa, V. , Ornelas, J. F. , Ramírez‐Barahona, S. , & Gándara, E. (2016). Historical reconstruction of climatic and elevation preferences and the evolution of cloud forest‐adapted tree ferns in Mesoamerica. PeerJ, 4, e2696 10.7717/peerj.2696 27896030 PMC5119233

[ece36189-bib-0086] Tovar‐Sánchez, E., Mussali‐Galante, P. , Esteban‐Jiménez, R. , Piñero, D. , Arias, D.M. , Dorado, O. , & Oyama, K. (2008). Chloroplast DNA polymorphism reveals geographic structure and introgression in the *Quercus crassifolia* x *Quercus crassipes* hybrid complex in Mexico. Botany‐Botanique, 86, 228–239. 10.1139/B07-128

[ece36189-bib-0087] Tovar‐Sánchez, E. , Valencia‐Cuevas, L. , Castillo‐Mendoza, E. , Mussali‐Galante, P. , Pérez‐Ruiz, R. V. , & Mendoza, A. (2013). Association between individual genetic diversity of two oak host species and canopy arthropod community structure. European Journal of Forest Research, 132, 165–179. 10.1007/s10342-012-0665-y

[ece36189-bib-0088] Valencia‐A, S. (1995). Contribución al conocimiento del género *Quercus* (Fagaceae) en el estado de Guerrero. Mexico: México Facultad de Ciencias, UNAM

[ece36189-bib-0089] Valencia‐A, S. (2004). Diversidad del género *Quercus* (Fagaceae) en México. Boletín De La Sociedad Botánica De México, 75, 33–53.

[ece36189-bib-0090] Valencia‐Cuevas, L. , Piñero, D. , Mussali‐Galante, P. , Valencia‐Ávalos, S. , & Tovar‐Sánchez, E. (2014). Effect of a red oak species gradient on genetic structure and diversity of *Quercus castanea* (Fagaceae) in Mexico. Tree Genetics and Genomes, 10(3), 641–652. 10.1007/s11295-014-0710-8

[ece36189-bib-0091] Villaseñor, J. L. , Delgadillo, C. , & Ortiz, E. (2006). Biodiversity hotspots from a multigroup perspective: mosses and senecios in the transmexican volcanic belt. Biodiversity and Conservation, 15, 4045–4058. 10.1007/s10531-005-3056-6

[ece36189-bib-0092] Watanabe, S. , Hajima, T. , Sudo, K. , Nagashima, T. , Takemura, T. , Okajima, H. , … Kawamiya, M. (2011). MIROC‐ESM 2010: Model description and basic results of CMIP5‐20c3m experiments. Geoscientific Model Development, 4, 845–872. 10.5194/gmd-4-845-2011

[ece36189-bib-0093] Wei, T. , & Simko, V. (2017). R package “corrplot”: Visualization of a Correlation Matrix (Version 0.84). Retrieved from https://github.com/taiyun/corrplot

